# Validation of Molecular Markers for Low Kunitz Trypsin Inhibitor Content in European Soybean (*Glycine max* L. Merr.) Germplasm

**DOI:** 10.3390/genes15081028

**Published:** 2024-08-05

**Authors:** Miroslav Bukan, Zoe Andrijanić, Ivan Pejić, Marko Ključarić, Lucija Čižmek, Ivana Tomaz, Nina Buljević, Hrvoje Šarčević

**Affiliations:** 1 Faculty of Agriculture, University of Zagreb, Svetošimunska Cesta 25, 10000 Zagreb, Croatia; mbukan@agr.hr (M.B.); marko.kljucaric136@gmail.com (M.K.); luce.cizmek@gmail.com (L.Č.); itomaz@agr.hr (I.T.); nbuljevic@agr.hr (N.B.); hsarcevic@agr.hr (H.Š.); 2 Centre of Excellence for Biodiversity and Molecular Plant Breeding (CroP-BioDiv), Svetošimunska Cesta 25, 10000 Zagreb, Croatia

**Keywords:** soybean, antinutrient, Kuntz trypsin inhibitor, marker-assisted selection

## Abstract

Trypsin inhibitors (TI) in raw soybean grain, mainly represented by the Kunitz trypsin inhibitor protein (KTI), prevent the normal activity of the digestive enzymes trypsin and chymotrypsin in humans and monogastric livestock. The inactivation of TI is achieved through costly and time-consuming heat treatment. Thermal processing also impairs the solubility and availability of the soybean grain protein. Therefore, the genetic elimination of KTI has been proposed as a suitable alternative to heat treatment. The aim of this study was to screen the collection of European soybean cultivars with six genetic markers (one SSR marker and five SNP markers) previously proposed as tightly linked to the *KTI3* gene encoding the major Kunitz trypsin inhibitor seed protein of soybean and validate their usability for marker-assisted selection (MAS). The six markers were validated on a subset of 38 cultivars with wide variability in KTI content and in the F_2_ and F_3:5_ progenies of two crosses between the known high- and low-KTI cultivars. Three genetic markers (SSR Satt228 and two SNP markers, Gm08_45317135_T/G and Gm08_45541906_A/C) were significantly associated with KTI content in a subset of 38 cultivars. Low-KTI alleles were detected in both low- and high-KTI genotypes and vice versa, high-KTI alleles were found in both high- and low-KTI genotypes, indicating a tight but not perfect association of these markers with the *KTI3* gene. The genetic marker SSR Satt228 showed a significant association with KTI content in the F_2_ progeny, while the SNP markers Gm08_45317135_T/G and Gm08_45541906_A/C allowed significant discrimination between progeny with high- vs. low-KTI progenies in the F_3:5_ generation. These three markers could be applied in MAS for low-KTI content but not without the additional phenotyping step to extract the desired low-KTI genotypes.

## 1. Introduction

In the last 60 years, the global area of soybean (*Glycine max* L. Merr.) cultivation has increased more than fivefold and is currently hitting its all-time record at around 130 million ha [1]. In the same period, the global soybean average grain yield has increased 2.6 times and recently reached a peak value of more than 2.6 tons per ha [1]. The growth in soybean production has been driven by the high demand for soybeans for processing and crushing. The majority of soybeans (approximately 76% by weight) are currently used as animal feed [2]. The high protein content (of about 40%) [3] makes soybeans the richest source of essential amino acids compared to other legumes and cereals and serves as the main protein feedstock for monogastric animals [4]. In addition to protein, soybean seeds also contain 30% carbohydrates and 20% fat (oil), while the remaining 10% consists of various functional substances, making soybeans a valuable food source for animals and humans [3]. Although many different health benefits are attributed to soybeans [5,6], only a relatively small percentage (about 6%) of soybean is used as food for human consumption, which is produced from whole soybeans and consumed in various processed forms [2]. Due to the high demand for protein meals and vegetable oils, global soybean production is expected to increase by 6% annually [6].

Despite the high content of nutritionally valuable compounds in soybean seed, soybean also contains some antinutritive compounds that have negative effects on human and animal health. Among these compounds, the proteinase inhibitors known as trypsin inhibitors (TI) are the most critical since they reduce protein digestibility in the human gut and monogastric livestock, i.e., they prevent normal activity of the digestive enzymes trypsin and chymotrypsin [7]. Two major forms of trypsin inhibitors in soybean seeds are the Kunitz trypsin inhibitor protein family (KTI) and the Bowman–Birk inhibitor protein family (BBI), which inhibit both trypsin and chymotrypsin [8]. KTI is much more abundant in soybean seeds than BBI and can account for up to 80% of total trypsin inhibitor activity [9]. For this reason, soybean seed has to be thermally treated before feeding, which efficiently degrades antinutritive TI compounds. However, heat treatment is costly, energy- and time-consuming, reduces the solubility of other soybean seed proteins, and decreases the availability of certain essential amino acids [10,11]. From an economic point of view, it would be preferable to use soybean seeds in their raw state as animal feed, similar to cereals and some other legumes. An alternative to the currently applied thermal treatment is the breeding of new cultivars with low TI content, as there is genetic variation for this trait [12,13,14,15,16]. In previous studies, 10 genes encoding KTI in soybeans were determined, but only four of them were transcribed into mRNA (*KTI1*, *KTI2*, *KTI3*, and *KTI4*) [17]. The *KTI3* gene was also found to encode the major Kunitz trypsin inhibitor seed protein, as its expression level is significantly higher in soybean seeds compared to other variants [17]. Analysis of the soybean genome revealed that a recessive null allele (*ti*− or *Kti−*) identified in the null KTI phenotype is caused by three frameshift mutations in the *KTI3* gene, which result in the premature termination of *KTI3* mRNA translation, resulting in a 100-fold reduction in *KTI3* mRNA in soybean embryos, i.e., KTI3*−* soybean lines lack Kunitz trypsin inhibitor activity [18]. The introgression of null alleles can reduce the KTI content in soybean seeds by 69–84% [5]. 

Besides the novel germplasm, fast and reliable methods for the assessment of the seed KTI content are equally important. The detection of KTI can be accomplished by several methods, including acrylamide sodium dodecyl sulphate polyacrylamide gel electrophoresis (SDS-PAGE), a standard colorimetric enzymatic assay that measures KTI activity, microfluidic or lab-on-a-chip technology, and two-dimensional liquid chromatography [15,19,20,21]. Rosso et al. [21] developed a high-performance liquid chromatography (HPLC) method as a high-throughput, cost-effective, and reliable assay to quantify KTI. Although the quantitative HPLC method is a very efficient and cost-effective method for measuring KTI content based on a very small number of seeds [21], it relies on seed samples and allows selection only after harvest.

Modern and efficient plant breeding is based on breeder-friendly genetic markers that can facilitate the detection of superior plants during the selection process. Having the tightly linked and codominant molecular markers to *KTI* loci could enable the selection of favorable homozygous F_2_ or F_3_ plants based on an extremely small amount of plant tissue. Kim et al. [11] used two bi-parental mapping populations with the aim of identifying DNA markers linked to the *KTI* locus. Eleven DNA markers (4 RAPD, 4 AFLP, and 3 SSR) used in their study were linked to the *KTI* locus. Of these, the SSR marker Satt228 was strongly linked within 0–3.7 cM of the *KTI* locus and was proposed for selection. This SSR marker was also confirmed to be reliable in the studies of Bulatova et al. [22] and da Silva et al. [23]. Recently, SNP markers have become the markers of choice. SNPs are highly abundant and repeatable and have proven useful in many applications, including marker-assisted selection for KTI [6,24]. Patil et al. [25] developed an SNP assay for the marker-assisted selection of various soybean seed composition traits, including KTI, and demonstrated efficient partitioning between homozygous and heterozygous F_2_ progenies of the cross ‘SP6A-206’ × ‘PI 542044’ (low KTI), but they omitted the phenotyping step in their study, which would prove that the markers used indeed discriminate between low and high KTI genotypes. Rosso et al. [24] developed a breeder-friendly SNP-KASP marker assay for low concentrations of Kunitz trypsin inhibitor in soybean, validated it in a set of 93 diverse soybean germplasm accessions, and identified three markers with selection efficiencies ranging from 31% to 86%.

The aim of this study was (1) to screen the germplasm collection managed and maintained by the Centre of Excellence for Biodiversity and Molecular Plant Breeding (CroP-BioDiv) using previously proposed SSR and SNP markers for marker-assisted selection of soybean KTI content and (2) validate these markers on soybean germplasm adapted to the growing conditions in southeastern Europe. 

## 2. Materials and Methods

### 2.1. Plant Material, Populations’ Development, and Choice of Markers

One hundred and sixty-five European soybean cultivars used in this study (Table S1) were part of the germplasm collection managed and maintained by the Centre of Excellence for Biodiversity and Molecular Plant Breeding (CroP-BioDiv, Zagreb, Croatia), and they were previously characterized and genotyped [26,27]. All cultivars were genotyped with molecular markers supposed to be tightly linked to the *KTI3* gene controlling KTI content: SSR locus Satt228 [11,22] and five SNP markers (Gm08_44265646_C/T, Gm08_44814503_C/T, Gm08_45270892_A/G, Gm08_45317135_T/G, and Gm08_45541906_A/C) proposed by Rosso et al. [24]. To validate the effect of these six markers on KTI content, a subset of 38 cultivars (Table S2) was selected from the entire collection of 165 cultivars.

To investigate the inheritance of KTI content and the usability of the proposed markers for MAS, two crosses were performed. Parents were selected primarily on the basis of KTI content in the seeds, but also considering other important agronomic traits. Two cultivars (‘Ascasubi’ and ‘Bahia’) developed and released by ERSA—the Regional Agency for Rural Development of Friuli Venezia Giulia (Udine, Italy)—were selected as sources of low KTI content. Two parent cultivars with high KTI content but also interesting agronomic traits from the breeder’s point of view were ‘DH_5170’ (developed by David Hendrick Sevita International, Canada, marketed by RWA Croatia) and ‘ES_Mentor’ (developed by Euralis Semences, France, marketed by Axereal, Nova Gradiška, Croatia). Two crosses ‘Ascasubi’ × ’DH_5170’ and ‘Bahia’ × ‘ES_Mentor’ were made in the 2018 season at the Department of Plant Breeding, Genetics and Biometrics of the University of Zagreb. In the 2020 season, leaf samples of F_2_ plants were collected for genotyping and seed samples were analyzed for KTI content by HPLC. In 2021, the F_3_ generation of both crosses was produced by single-seed descent. The seeds of the F_3_ plants were bulked and advanced to F_3:5_ generation in 2021 and 2022. After selection in the field in 2023, a sample of promising F_3:5_ families of the crosses ‘Ascasubi’ × ’DH_5170’ and ‘Bahia’ × ‘ES_Mentor’ (seed mixture of 20 plants per family) was analyzed for the KTI seed content and additionally genotyped with the SNP markers Gm08_45317135_T/G and Gm08_45541906_A/C [24] using the KASP assay.

### 2.2. Satt228 Genotyping 

DNA was isolated from the leaf samples of 165 soybean cultivars as well as from 62 and 54 F_2_ plants of the crosses ‘Ascasubi’ × ‘DH_5170’ and ‘Bahia’ × ‘ES_Mentor’, respectively, using the peqGold DNA Plant Mini Kit according to the PeqLab protocol for plant DNA isolation (PEQLAB Biotechnologie GmbH, Erlangen, Germany).

The PCR reaction was performed in a Veriti thermocycler (Applied Biosystems, Foster City, CA, USA) in a total volume of 10 µL. The following components were added to the reaction mixture: 10 ng of template DNA of each sample, 0.3 µM of forward primer (TCATAACGTAAGAGATGGTAAAACT) and reverse primer (CATTATAAGAAAACGTGCTAAAGAG), and 200 µM of each dNTP, 2 mM MgCl2, 0.75 U Taq polymerase (Sigma-Aldrich, Hamburg, Germany), 0.5X CES PCR Amplifier, and 1X PCR Buffer. The following thermal cycling protocol was applied: pre-cycle at 94 °C for 2 min; 30 cycles of denaturation for 1 min at 94 °C, 45 s of annealing at 49 °C and 45 s extension at 72 °C; post-cycle of 10 min at 72 °C; and then termination at 4 °C. PCR products were detected by capillary electrophoresis using an ABI 3130 Genetic Analyzer (Applied Biosystems, Foster City, CA, USA) with a GeneScan-500 LIZ size standard (Thermo Fisher Scientific, Waltham, MA, USA). GeneMapper 4.0 software (Applied Biosystems, Foster City, CA, USA) was used to detect allele sizes expressed as the number of base pairs (bp).

### 2.3. SNP Genotyping 

SNP profiles at five SNP markers (Gm08_44265646_C/T, Gm08_44814503_C/T, Gm08_45270892_A/G, Gm08_45317135_T/G, and Gm08_45541906_A/C) for 165 soybean cultivars were extracted from the whole-genotype profiles containing 52,041 SNPs obtained by prior genotyping of 207 soybean cultivars on the SoySNP50K Illumina Infinium BeadChip (Illumina, Inc., San Diego, CA, USA) by Andrijanić et al. [27].

### 2.4. KASP Genotyping 

Samples of young leaves from five plants within each of the F_3:5_ families of the crosses ‘Ascasubi’ × ‘DH_5170’ and ‘Bahia’ × ‘ES_Mentor’ were collected using the BioArk™ Leaf collection kit (LGC, Biosearch Technologies™, Hoddesdon, UK). The leaf samples along with the sequence information for two SNP markers (Gm08_45317135_T_G and Gm08_45541906_A_C) were sent to LGC Biosearch Technologies for DNA extraction and KASP genotyping conducted within all-inclusive service [28].

### 2.5. Measurement of KTI Content 

KTI content was measured in a subset of 38 cultivars, in seed samples of F_2_ plants from the crosses ‘Ascasubi’ × ’DH_5170’ and ‘Bahia’ × ‘ES_Mentor’, and in the seed samples of the F_3:5_ lines of the two mentioned crosses. The analysis of KTI was conducted according to the method described by Rosso et al. [21]. In brief, 10 mg of soybean seed powder was mixed with 1.5 mL of 0.1 M sodium acetate buffer (pH 4.5). The samples were stirred at room temperature for 1 h and then at 14,000 rpm for 15 min. The obtained supernatant was filtered through a syringe with an IC Millex-LG 0.2 µm membrane filter (Merck Millipore LTD., Carrigtwohill, Ireland). The separation, identification, and quantification of KTI were performed using a Vanquish Flex UHPLC system (Thermo Fisher Scientific, Waltham, MA, USA). Separation was performed with a Poros R2/H perfusion analytical column (2.1 × 100 mm, 10 µm) heated at 60 °C. The solvents consisted of 0.01% (*v*/*v*) trifluoroacetic acid in Milli-Q water (eluent A) and 0.085% (*v*/*v*) trifluoroacetic acid in acetonitrile (eluent B), and the flow rate was 1.0 mL/min. The linear gradient for eluent B was as follows: 0 min, 17%; increasing to 22% within 3.5 min, to 35% in the following 0.5 min, to 41% within another 6 min, then to 95% in 0.5 min, and finally returning to 17% in 0.5 min. The injection volume for each sample was 10 μL while the detection wavelength was 220 nm. Quantification was performed using the external standard method.

### 2.6. Statistical Analyses

The agreement of the observed segregation ratio at the SSR locus Satt228 in the F_2_ generation of the crosses ‘Ascasubi’ × ‘DH_5170’ and ‘Bahia’ × ‘ES_Mentor’ with the expected ratio of 1:2:1 in the context of Mendelian monogenic inheritance was tested using the chi-square (χ^2^) test. The difference between the genotypic means for the KTI content in the F_2_ generation of the two crosses was compared using the Fisher LSD test following the general linear model (GLM) procedure. The mean values of the two homozygous allele classes at two SNP loci in the F_3:5_ families of the crosses ‘Ascasubi’ × ‘DH_5170’ and ‘Bahia’ × ‘ES_Mentor’ and at six loci (SSR locus Satt228 and five SNP loci) in the validation set of 38 cultivars were compared using the *t*-test. All statistical analyses were performed using SAS/STAT version 9.4 [29].

## 3. Results

### 3.1. Allele Frequencies at Marker Loci in the Cultivar Panel

**Figure 1:**
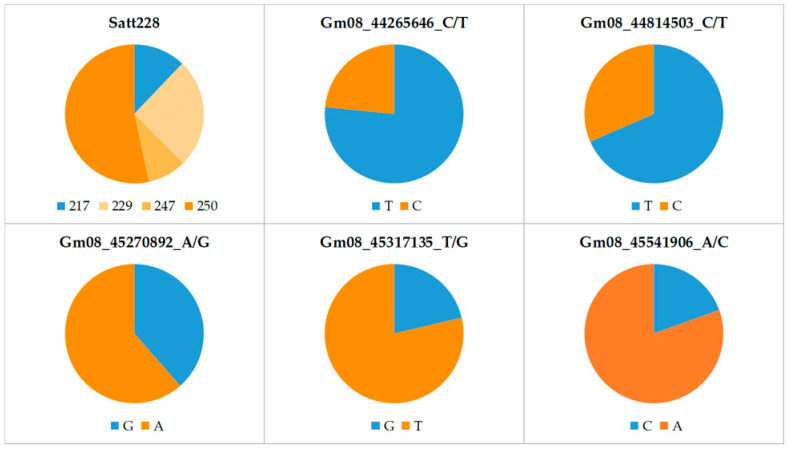
Allele frequencies at six marker loci associated with KTI content in the 165 soybean cultivars. Blue and orange colors indicate low- and high-KTI alleles, respectively.

The allele frequencies at six marker loci in the panel of 165 soybean cultivars are shown in Table S1 and Figure 1. For the SSR locus Satt228, the highest frequency was observed for allele 250 (53%) and the lowest for allele 247 (9%). The frequency of the low-KTI allele (217) was only 12% at this locus. In contrast, at the SNP loci Gm08_44265646_C/T and Gm08_44814503_C/T, the low-KTI allele was found in 77% and 68% of the cultivars, respectively. A relatively high frequency of the low-KTI allele was also found at the SNP locus Gm08_45270892_A/G (39%). In contrast, at the two remaining SNP loci, Gm08_45317135_T/G and Gm08_45541906_A/C, the low-KTI alleles had considerably lower frequencies (21% and 19%, respectively).

**Figure 2:**
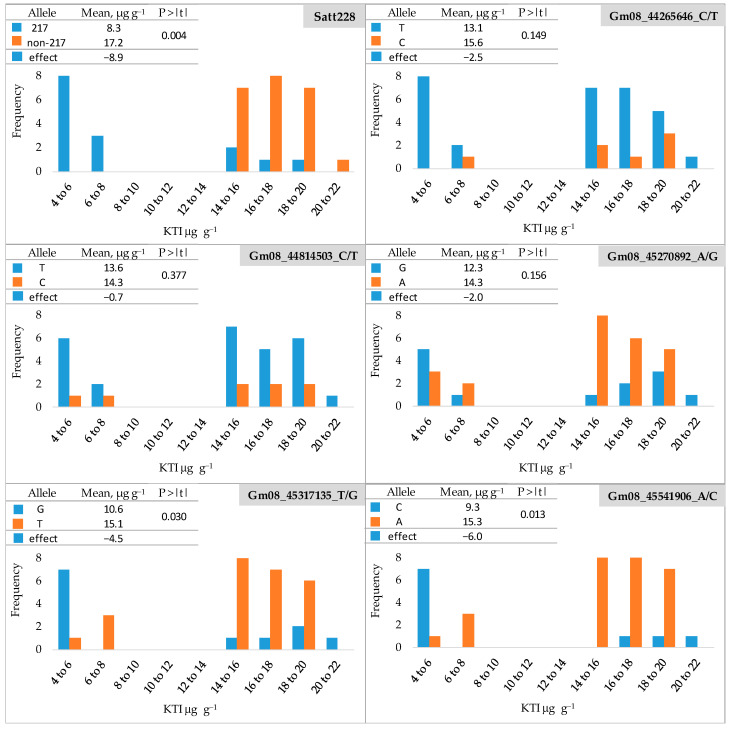
Frequency distribution of KTI content (µg g^−1^) in seeds of 38 soybean cultivars for low- and high-KTI alleles at six marker loci. Blue and orange bars indicate low- and high-KTI alleles, respectively. P > |t|—the significance of the *t*-test.

The frequency distribution of KTI content (µg g^−1^) in the seeds of 38 soybean cultivars (a subset of 165 cultivars) for low- and high-KTI alleles at six marker loci is shown in Figure 2 and Table S2. The soybean cultivars were clearly divided into two groups with a KTI content ranging from 4.50 to 6.56 µg g^−1^ in the low-KTI group and from 14.07 to 21.40 µg g^−1^ in the high-KTI group. Regarding the SSR locus Satt228, all low-KTI cultivars had the low-KTI allele (217). The high-KTI cultivars carried predominantly high-KTI alleles (non-217), with a proportion of low-KTI allele of 15% (Table S3). The mean KTI content of the cultivars with high-KTI alleles (non-217) was 17.2 µg g^−1^ and was significantly reduced by 8.9 µg g^−1^ (52%) in the cultivars with the low-KTI allele (217). 

For the SNP loci Gm08_44265646_C/T) and Gm08_44814503_C/T), all but one and two cultivars in the high-KTI group, respectively, carried the low-KTI allele (T) (Figure 2), resulting in the proportions of low-KTI alleles of 91 and 80%, respectively (Table S3). However, the low-KTI alleles were also overrepresented in the high-KTI group, with the proportions of 77 and 76% for Gm08_44265646_C/T and Gm08_44814503_C/T, respectively (Table S3). For these two SNP markers, no significant differences were found between the mean values of the group with a low-KTI allele (T) and the group with a high-KTI allele (C) (Figure 2). At the SNP locus Gm08_45270892_A/G (Figure 2), the low-KTI allele (G) was found with a proportion of 55% and 27% in the low-KTI and high-KTI cultivar groups, respectively (Table S3). For this SNP marker, the difference between the mean values of the low-KTI allele group (G) and the high-KTI allele group (A) was also not significant (Figure 2).

For the two remaining SNP loci Gm08_45317135_T/G and Gm08_45541906_A/C, a similar distribution of low- and high-KTI alleles was observed between the groups, with low-KTI alleles being more common in the low-KTI cultivars and high-KTI alleles prevailing in the high KTI group. The proportion of low-KTI alleles in the low-KTI group was 64% for both markers, while in the high-KTI group, it was 19 and 11% for Gm08_45317135_T/G and Gm08_45541906_A/C, respectively (Table S3). The low-KTI allele significantly reduced the KTI content by 4.5 µg g^−1^ (30%) and 6.0 µg g^−1^ (39%) for the markers Gm08_45317135_T/G and Gm08_45541906_A/C, respectively (Figure 2).

### 3.2. SSR Validation in F_2_

The segregation analysis of the F_2_ generations of the crosses ‘Ascasubi’ × ‘DH_5170’ and ‘Bahia’ × ‘ES_Mentor’ for the SSR locus Satt228 is shown in Table 1. The χ^2^-test revealed no significant deviation between the observed segregation of the three genotypes and the expected segregation, confirming Mendelian monogenic inheritance in both F_2_ populations studied.

**Figure 3:**
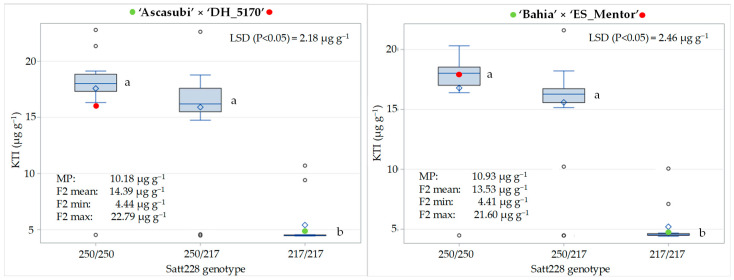
Distribution of KTI content (µg g^−1^) for three genotypic classes (250/250, 250/217, and 217/217) at SSR locus Satt228 in F_2_ generation of the crosses ‘Ascasubi’ × ‘DH_5170’ and ‘Bahia’ × ‘ES_Mentor’. Differences among genotype means followed by the same letter (indicated next to box plots) are not significantly different at *p* < 0.05 according to the Fisher LSD test. Means of low-KTI and high-KTI parents are indicated with green and red circles, respectively. MP indicates midparent value.

The KTI content varied from 4.44 to 22.79 µg g^−1^ in the F_2_ of the cross ‘Ascasubi’ × ‘DH_5170’ and from 4.41 to 21.60 µg g^−1^ in the F_2_ of the ‘cross ‘Bahia’ × ‘ES_Mentor’, with a corresponding mean value of 14.39 and 13.53 µg g^−1^ (Figure 3). The mean KTI content of the parents with low KTI content was 4.52 µg g^−1^ (‘Ascasubi’) and 4.50 µg g^−1^ (‘Bahia’) and that of the parents with high KTI content was 15.84 µg g^−1^ (DH_5170) and 17.36 µg g^−1^ (‘ES_Mentor’). A significantly higher F_2_ mean compared to the midparent value (MP) for both crosses is consistent with the dominant inheritance of the KTI3 gene, with high-KTI allele homozygotes (250/250) and heterozygotes (250/217), which together account for three-quarters of the total progeny, having a significantly higher KTI content than low-KTI allele homozygotes (217/217). The difference between the F_2_ mean and the MP was greater in the cross ‘Ascasubi’ × ‘DH_5170’ than in ‘Bahia’ × ‘ES_Mentor’, reflecting a pronounced negative transgressive segregation observed in the first cross in the class of high-KTI allele homozygous genotypes. In the ‘Ascasubi’ × ‘DH_5170’ cross, the mean KTI content in the high- and low-KTI allele homozygotes was 17.58 µg g^−1^ and 5.41 µg g^−1^, respectively, while the corresponding KTI content in the ‘Bahia’ × ‘ES_Mentor’ cross was 16.69 µg g^−1^ and 5.52 µg g^−1^, respectively, resulting in a 69% and 67% reduction in KTI content due to the effect of the low-KTI allele in the two crosses.

### 3.3. SNP Validation in F_3:5_ Families

**Figure 4:**
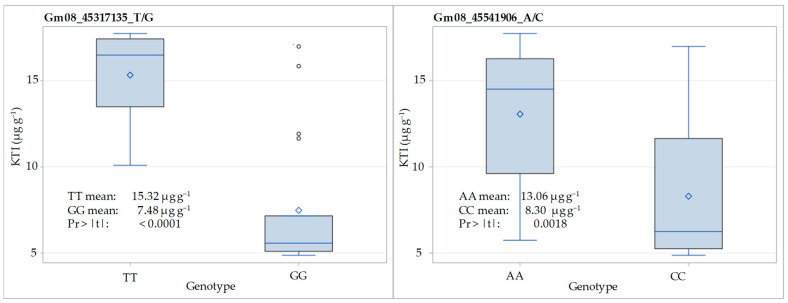
Distribution of KTI content (µg g^−1^) for homozygous genotypes at SNP loci Gm08_45317135_T/G and Gm08_45541906_A/C in F_3:5_ of the crosses ‘Ascasubi’ × ‘DH_5170’ (*N* = 12) and ‘Bahia’ × ‘ES_Mentor’ (*N* = 32). Pr > |t| indicates the significance of the *t*-test.

The distribution of KTI content (µg g^−1^) for homozygous genotypes at the SNP loci Gm08_45317135_T/G and Gm08_45541906_A/C in the F_3:5_ generation of the crosses ‘Ascasubi’ × ‘DH_5170’ and ‘Bahia’ × ‘ES_Mentor’ is shown in Figure 4. Regarding the locus Gm08_45317135_T/G, the KTI content varied from 10.09 to 17.73 µg g^−1^ in TT homozygotes and from 4.87 to 16.98 in GG homozygotes, with corresponding means of 15.32 and 7.48 µg g^−1^. A significant reduction in KTI content due to low-KTI allele (G) was 51%. With respect to the locus Gm08_45541906_A_C, the KTI content varied from 5.74 to 17.73 µg g^−1^ in AA homozygotes and from 4.87 to 16.98 in CC homozygotes, with corresponding means of 13.06 and 8.30 µg g^−1^. The significant reduction in KTI content due to low-KTI allele (C) was 37%.

## 4. Discussion

The six genetic markers, one SSR—Satt228 [11,22] and five SNP markers, Gm08_44265646_C/T, Gm08_44814503_C/T, Gm08_45270892_A/G, Gm08_45317135_T/G, and Gm08_45541906_A/C [24], which have been previously identified as tightly linked to the *KTI3* gene on chromosome 8 of soybean, were applied to characterize the panel of 165 European soybean cultivars within the Croatian Centre of Excellence for Biodiversity and Molecular Plant Breeding (CroP-BioDiv). The SSR marker Satt228 and two SNP markers (Gm08_45317135_T/G and Gm08_45541906_A/C) were within the examined set of cultivars represented mostly with their high-KTI alleles (88%, 81%, and 82% of non-217 alleles, T alleles, and A alleles, respectively), as expected in the panel of cultivars, which were generally not bred for low-KTI content. On the other hand, two SNP markers, Gm08_44265646_C/T and Gm08_44814503_C/T, were mainly represented by their low-KTI alleles (76 and 68% of T alleles detected, respectively). The representation of the low-KTI allele (G) of the SNP marker Gm08_45270892_A/G was also relatively common at 38%. 

The same six genetic markers were used for validation in a sub-sample of 38 cultivars (derived from a set of 165 screened cultivars). The mean KTI content of cultivars with low-KTI alleles at the three markers, SSR Satt228 and SNPs Gm08_45317135_T/G and Gm08_45541906_A/C, was significantly reduced compared to cultivars possessing high-KTI alleles (Figure 2). Within the three markers, the proportion of low-KTI alleles within the low-KTI cultivars was 64% for the two SNP markers (alleles G and C of Gm08_45317135_T/G and Gm08_45541906_A/C, respectively), and 100% for the low-KTI allele 217 of the Satt228 SSR marker (Table S3). For the two SNP markers, this also indicates that 36% of cultivars characterized by low-KTI content would be missed since they possessed high-KTI marker alleles, i.e., they would not end up being selected in the strict marker-assisted selection. For the remaining three SNP markers, Gm08_44265646_C/T, Gm08_44814503_C/T, and Gm08_45270892_A/G, no significant differences in KTI content between cultivars carrying low- or high-KTI alleles were observed. These three SNP markers were, in the study of Rosso et al. [24], validated in 93 plant introductions (PIs) with low and high KTI content. Rosso et al. [24] discussed the representation of the low-KTI alleles of these three SNP markers as selection efficiency only within the PIs exhibiting low KTI content and found their selection efficiency to be 64%, 86%, and 31%, respectively. However, our results show that the selection of the low-KTI allele would result in the selection of an additional number of high-KTI genotypes since the low-KTI alleles were also present within the group of genotypes with high KTI content. For example, for SNP marker Gm08_44814503_C/T, which, in the study by Rosso et al. [24], showed the best selection efficiency (86%), the low-KTI allele in the present study was found in 80% of cultivars with low KTI content (Table S3), which is consistent with the results of Rosso et al. [24]. However, the low-KTI allele was represented by a similar proportion (76%) among the cultivars with high KTI content, making this marker inefficient in discriminating the two cultivar groups. On the other hand, the representation of the low-KTI alleles of the three efficient markers among the cultivars with high KTI content was much lower in the present study, reaching 15%, 19%, and 11% for SSR Satt228, Gm08_45317135_T/G, and Gm08_45541906_A/C, respectively. The reason for the discrepancy between the specific marker allele and the KTI content likely lies in the fact that these markers are tightly but not perfectly linked to the *KTI3* gene and that the efficiency of the specific marker also varies upon the genetic background of the germplasm. Previous studies have identified several genes associated with Kunitz trypsin inhibitors on chromosome 8 of soybean, with *KTI3* being only one of them [24].

In the present study, we focused on the KTI content in soybean seeds. However, according to Rosso et al. [24], breeding only for low or null KTI content in soybean would not be sufficient for practical applications because of the effect of environmental conditions on the trait, the interaction between the environment and the genotype, the expression of 1 of the 13 isoforms of the *KTI3* gene in seeds, and the presence of Bowman–Birk TI, which affects TI activity in soybean seeds. In addition, Gillman et al. [15] detected a similar reduction in relative trypsin and chymotrypsin activity in the KTI1−, KTI3−, and KTI1/3− genotypes, with the lowest value of detected activity in the KTI1/3− genotype. This indicates that selection for the low-KTI allele of the *KTI1* gene can be equally efficient in reducing total TI content, regardless of the fact that *KTI3* is the dominantly expressed isoform of the *KTI* gene in soybean seeds. Wang et al. [30] also demonstrated a significant reduction in KTI content in KTI1-mutated soybean lines developed by CRISP/Cas-mediated mutagenesis, although the highest reduction in total KTI content was achieved in the genotypes with knockout mutations on both *KTI1* and *KTI3* genes, similar to Gillman et al. [15]. The important question to be asked is whether the reduction achieved in KTI content, and consequently, the total TI content, is sufficient from a practical point of view to allow the use of raw soybean seeds and/or their derivatives in animal feed. Chickens fed crude soybean extract obtained from a cultivar with low levels of KTI did not have inhibited growth compared to chickens fed heat-treated soybeans that had a standard level of KTI [31]. In contrast, Perić et al. [32] found a lower level of performance in the group of pigs fed raw low-KTI soybean in comparison to pigs fed extruded standard and extruded low-KTI soybean. Similarly, Gillman et al. [15] reported that weight gain for young animals fed non-heat-treated soybean materials (including KTI3−) is inferior to those fed conventional heat-treated soybeans. Gillman et al. [15] indicated that developing seeds of KTI− lines actively pursue TI protein homeostasis through two different methods: elevated BBI mRNA levels (KTI1−) or proteome rebalancing (KTI3−), although the exact biochemical mechanisms are still not completely understood. The same authors also noted that a substantial reduction in trypsin/chymotrypsin inhibitors, beyond that currently possible with the KTI1− and/or KTI3− mutations, would be necessary to allow the use of raw soybean derivatives without the pancreatic complications or impaired weight gain issues of conventional soybean seed. This highlights the necessity of searching for new germplasms with new non-functional KTI mutants and pyramiding already-known genes and also highlights the complexity of breeding soybean genotypes with low TI activity.

The SSR locus Satt228 has been identified to be tightly linked to the *TI3* (or *KTI3*) locus [11]. In our study, we performed two crosses (‘Ascasubi’ × ‘DH_5170’ and ‘Bahia’ × ‘ES_Mentor’), each involving one low-KTI parent (‘Ascasubi’, ‘Bahia’) and one high-KTI parent (‘DH_5170’ and ‘ES_Mentor’). The SSR locus Satt228 clearly distinguished the low-KTI homozygotes (characterized by the low-KTI allele 217) from the high-KTI homozygotes (non-217 alleles) and heterozygotes. As reported by Bulatova et al. [22], who crossed the Kazakh high-KTI cultivar ‘Lastochka’ with two low-KTI Italian cultivars ‘Ascasubi’ and ‘Hilario’, the observed segregation ratios in the F_2_ progeny did not deviate from the expected Mendelian inheritance pattern. In contrast to Bulatova et al. [22], in our study, we presented the KTI protein content for all three genotype classes (low-KTI allele homozygotes and high-KTI alleles homozygotes and heterozygotes) and the Satt228 locus showed a clear dominance inheritance pattern, i.e., genotypes possessing high-KTI SSR alleles (227, 247, 250) in the homozygous and heterozygous (paired with the low-KTI allele 217) state were characterized by a similar and significantly higher KTI content than low-KTI homozygotes. In our study, the reduction in KTI content, which could be attributed to the effect of the low-KTI allele (217) in the F_2_ progeny, was 69% (for the cross ‘Ascasubi’ × ‘DH_5170′) and 67% (for the cross ‘Bahia’ × ‘ES_Mentor’), in comparison to the high-KTI and low-KTI homozygotes. This magnitude of reduction was higher than in Bulatova et al. [22], who reported a 45% and 62% reduction in KTI activity in the low-KTI allele homozygotes compared to the high-KTI allele homozygotes in the F_3_ populations of the crosses between the high-KTI cultivar ‘Lastochka’ and the low-KTI cultivars ‘Ascasubi’ and ‘Hilario’, respectively. In the study by Kumar et al. [5], the reduction in trypsin inhibitor content (compared to the recurrent parent ‘JS97-52′) varied from 68.76 to 83.52% in the nine developed low-KTI introgression lines.

In our study, we validated two SNP markers, Gm08_45317135_T/G and Gm08_45541906_A/C, in the F_3:5_ families of the crosses ‘Ascasubi’ × ‘DH_5170’ and ‘Bahia’ × ‘ES_Mentor’ and found a reduction in KTI content of 51% and 37%, respectively, for the two markers in the low-KTI homozygotes compared to the high-KTI allele homozygotes. The two SNP markers investigated were significantly associated with the concentration of KTI in the mapping population derived from the ‘Glenn’ × ‘PI 547656’ cross in the study of Rosso et al. [24], and the same was confirmed in the present study in a set of 38 cultivars. 

## 5. Conclusions

Three of the six markers for low KTI content in soybean seed, validated in the present study, showed a significant association with KTI content in a panel of European soybean cultivars. These markers also proved to be efficient in the selection of progenies with low KTI content in the segregating generations of crosses involving parents with contrasting KTI genotypes/phenotypes. In this regard, the SSR marker Satt228 proved to be more efficient than the two SNP markers Gm08_45317135_T/G and Gm08_45541906_A/C. However, regardless of marker type, our results suggest that selection for low KTI content based on the desired marker allele should be followed by additional phenotyping to confirm the desired low-KTI genotypes in the selection of parents for crosses and in the verification of low-KTI content for homozygous progeny in segregating generations. Nevertheless, the present study confirmed that the use of markers increases the efficiency of selection for low KTI by reducing the number of lines that require costly additional phenotyping. The present study, as well as most of the previous studies, deals with markers for the *KTI3* gene, which is a major gene, but not the only gene, controlling the KTI content in soybean seeds. Therefore, the inclusion of newly developed markers for other *KTI* genes and their validation in germplasms of different genetic backgrounds together with the exploration of environmental influences on KTI content would provide deeper insights into the effectiveness of MAS for low KTI content in soybean.

## Figures and Tables

**Table 1:** Chi-square (χ^2^) test for segregation of genotypes at the SSR locus Satt228 in F_2_ generation of ‘Ascasubi’ × ‘DH_5170’ and ‘Bahia’ × ‘ES_Mentor’ crosses.

	Satt228 Genotype			χ^2^	DF	Pr > χ^2^
	250/250	250/217	217/217			
Cross		‘Ascasubi’ × ‘DH_5170′				
Observed segregation	12	31	19	1.5806	2	0.4537
Expected segregation	15.5	31	15.5			
Cross		‘Bahia’ × ‘ES_Mentor’				
Observed segregation	14	28	12	0.2222	2	0.8948
Expected segregation	13.5	27	13.5			

Pr > χ^2^—significance of the chi-square test.

## Data Availability

The original contributions presented in the study are included in the article/Supplementary Materials. Further inquiries can be directed to the corresponding authors.

## Supplementary Materials

The following supporting information can be downloaded at: https://www.mdpi.com/article/10.3390/genes15081028/s1, Table S1: Marker profiles of 165 soybean cultivars; Table S2: KTI content and profile at six marker loci for 38 soybean cultivars; Table S3: Proportion of low-KTI allele (%) within the low- and high-KTI cultivar group at six marker loci.
